# Rapid and Precise Semi-Automatic Axon Quantification in Human Peripheral Nerves

**DOI:** 10.1038/s41598-020-58917-4

**Published:** 2020-02-06

**Authors:** S. Engelmann, M. Ruewe, S. Geis, C. D. Taeger, M. Kehrer, E. R. Tamm, R. L. A. W Bleys, F. Zeman, L. Prantl, A. Kehrer

**Affiliations:** 1 Department of Plastic, Hand and Reconstructive Surgery, University Hospital Regensburg, Regensburg, Germany; 2 Department of Trauma Surgery, University Hospital Bonn, Bonn, Germany; 3 Institute of Human Anatomy, University of Regensburg, Regensburg, Germany; 4 Department of Anatomy, University Medical Center Utrecht, Utrecht, The Netherlands; 5 Center for Clinical Studies, University Hospital Regensburg, Regensburg, Germany

**Keywords:** Cellular imaging, Peripheral nervous system, Peripheral nervous system

## Abstract

We developed a time-efficient semi-automated axon quantification method using freeware in human cranial nerve sections stained with paraphenylenediamine (PPD). It was used to analyze a total of 1238 facial and masseteric nerve biopsies. The technique was validated by comparing manual and semi-automated quantification of 129 (10.4%) randomly selected biopsies. The software-based method demonstrated a sensitivity of 94% and a specificity of 87%. Semi-automatic axon counting was significantly faster (p < 0.001) than manual counting. It took 1 hour and 47 minutes for all 129 biopsies (averaging 50 sec per biopsy, 0.04 seconds per axon). The counting process is automatic and does not need to be supervised. Manual counting took 21 hours and 6 minutes in total (average 9 minutes and 49 seconds per biopsy, 0.52 seconds per axon). Our method showed a linear correlation to the manual counts (R = 0.944 Spearman rho). Attempts have been made by several research groups to automate axonal load quantification. These methods often require specific hard- and software and are therefore only accessible to a few specialized laboratories. Our semi-automated axon quantification is precise, reliable and time-sparing using publicly available software and should be useful for an effective axon quantification in various human peripheral nerves.

## Introduction

Microscopic analysis of peripheral nerves is key for many clinical and research based projects. Peripheral nerves have been analyzed through multiple methods, which can generally be categorized into ‘manual’, ‘automated’ and ‘semi-automated’ methods. Here, the terms for ‘manual’ and ‘fully automated’ morphometry will be used as previously described^1–4^. ‘Semi-automated’ will be used synonymously with Urso-Baiardas ‘interactive automated’ approach; an automated method with the opportunity for manual preparation or alteration^3^.

In the past, no prime and uniform method could be found, that is simple, cost efficient and time sparing. Therefore, tendentially, small research collectives use manual methods for analysis^1,2,5^. Attempts have been made by several research groups throughout medical and scientific research, to automate this process^3,6–8^. Unfortunately, it is often found, that these methods are either highly specialized, thus accessible to only few expert laboratories, or, in the case of highly developed software and hardware, very costly^3,9^. By example, Marina *et al*. have coined a method which, similar to this project, focuses on simpler semi-automated anaylsis^10^. Other research groups such as Hunter *et al*. focus on highly specialized methods, which are able to produce a wide range of data and process numerous variables^11^.

The semi-automated quantification method proposed in this study was developed as part of a greater study on Human facial nerves, for which a time sparing, cost efficient and user-friendly method of axonal quantification was required. Patients with facial palsy, caused by dysfunction of the seventh cranial nerve, suffer emotional distress and are often socially isolated^12^. The treatment of irreversible facial palsy remains a special challenge for reconstructive surgery. A popular and feasible reconstructive method is a Cross-face-nerve-graft (CFNG). Here motor axon capacity is diverted from the sane facial half to the paralyzed via sural nerve grafts coapted to adequate branches of the functional facial nerve. Terzis *et al*. and several other facial surgeons have shown that the surgical outcome correlates to axon load of the donor nerve^13–18^. Consequently Terzis determined an axonal capacity of 900 as the cut off for good functional outcomes^13^. In peripheral facial nerve surgery axon quantities of interest seldomly exceed 2000^13,19^. To the best of our knowledge, such a cut off value, has not yet been investigated in other reconstructive nerve procedures. However, it is known that axonal loads in peripheral nerve reconstruction, such as brachial plexus surgery, are similar to those seen in facial nerve surgery^20^.

Kehrer *et al*. designed a large macro and microscopic study to further examine anatomical characteristics of the seventh cranial nerve^21,22^. Further morphometry of the fifth cranial nerve was executed (unpublished data). Within this study axon quantification of 1238 nerve biopsies had to be performed. Quantifying axon capacity is a key part of the study, especially for determining a cut off axon count of 900 axons within a facial nerve branch.

Evaluation of preexisting methods for nerve fibre analysis and axon quantification led us to set the goal to develop a refined method of axon quantification fulfilling several requirements. The novel method must incorporate a fully automated component with stack evaluation for fast processing of large study cohorts, a semi-automated component to allow adjustments and corrections, the use of freeware for cost-efficiency, reproducibility and adequate accuracy with exact counts and value margins.

## Methods

Preparation for nerve fibre analysis in this study can be briefly structured into different steps. Body donor microsurgical dissection was executed under 4x loupe magnification and followed by extraction of nerve biopsies at up to 19 different anatomical locations of respective branches throughout the extracranial course of facial and masseteric nerves. Biopsies were fixated and further processed for histology. The probes were micro cut, stained and prepared as microscope slides. Nerve transections were then digitalized. The digital image of each nerve biopsy transection was used for further morphometry processing. A collective of 129 biopsies from the total of 1238 nerve biopsies was randomly selected for counting. Both manual and semi-automatic counts were carried out using the Fiji-freeware. All methods were performed in accordance with relevant guidelines and regulations. The study was approved by the institutional ethics review board (Ethics Committee of the University Regensburg, Germany). A step-by-step protocol for the whole process can be found in the Supplementary Data.

### Anatomical dissection

106 fresh frozen cadaver facial halves were dissected from 6/2015 to 9/2016 at departments of anatomy of the University of Regensburg/Germany, University of Halle/Germany, University Medical Center Utrecht/Netherlands and University of Graz/Austria. Nerve specimens were obtained only from body donors who entered the according anatomical institute through a donation program with written informed consent during life time. Institutional ethical review board approval was obtained. (*File reference number 14-101-0251, Ethics Committee of the University Regensburg, Germany)*. A detailed anatomical dissection of the facial halves was carried out as described previously^23^.

### Extraction of nerve specimens

**Figure 1:**
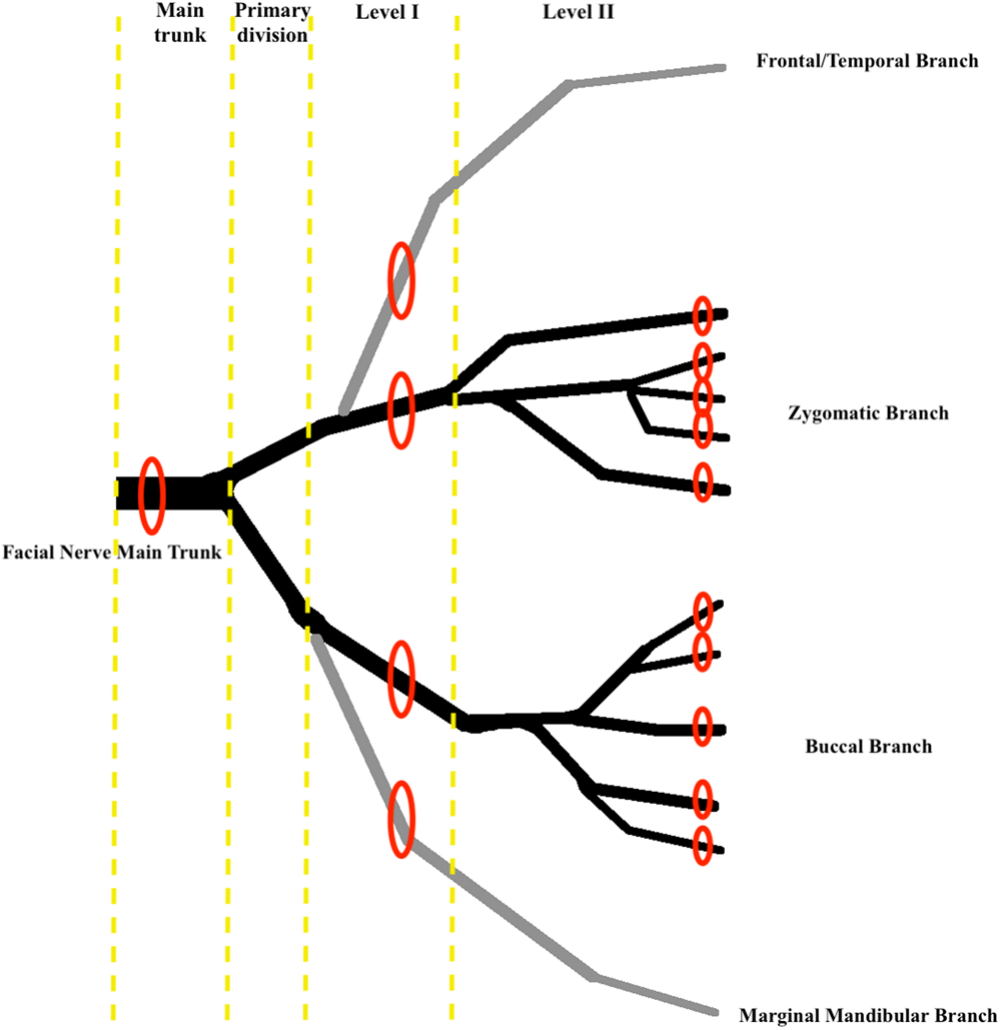
shows a schematic illustration of the peripheral extracranial facial nerve, its relevant branches and biopsy sites (red ellipses). The yellow lines indicate topographic levelling.

Nerve specimens were obtained at clinically relevant anatomical locations throughout the extracranial course of the facial and masseteric nerve. This included the extratemporal facial nerve main trunk 1 cm peripheral from its exit through the stylomastoid foramen, a temporofrontal main branch, a marginal mandibular main branch, zygomatic and buccal main branches, as well as branches of the zygomatic and buccal nerve systems. Branches touching the zygoma or lying topographically superficial to it were defined as zygomatic, branches inferior to the zygoma were defined as buccal. Specimens were taken at two different levels distal to the primary division of the facial nerve bifurcating into a temporofacial (‘upper division’) and cervicofacial (‘lower division’). Level I was defined as direct branches of the primary division. Level II as branches of level I branches^24^. (Fig. 1)

In addition, probes of the main trunk of the masseteric nerve just lateral to its course through the incisura mandibulae and its distal horizontal and vertical branches were taken. Detailed description of facial nerve branching and anatomical definitions of zygomatic and buccal branches was recently posed by Kehrer *et al*.^24^. Figure 1 shows a simplified illustration of different nerve specimen locations. After dissection and extraction the nerve biopsies were directly processed for histological analysis.

### Fixation and embedding

Nerve biopsies were set into labelled Eppendorf tubes and fixed overnight in an EM-fixative solution (2.5% formaldehyde, 2.5% glutaraldehyde) modified after Ito and Karnovsky^25^.

After a 24 hours fixation period the specimens were washed with sodium cacodylate buffer at pH 7.4 four times for 30 minutes. The samples were then osmicated for 2 hours and 30 minutes at 4 °C in a solution of 1% OsO_4_ + 0.8% potassium hexacyanoferrate II. Subsequently, samples were washed with bidistilled water four times for 30 minutes and left overnight. The samples were then serially dehydrated in alcohol and impregnated with epoxy resin. Finally, the labelled samples were embedded in rubber moulds with epoxy resin for 24 hours at 60 °C^21^. After removal from the rubber moulds, the resin blocks were ready for histologic sectioning.

### Production of microscope slides with semi thin transection cuts

1 µm thick sections were cut using an ultramicrotome (LKB, Sweden) and a diamond edge knife (Diatome, Switzerland). Cuts for each specimen were collected in a knife-mounted bath of bidistilled water, then transferred to a glass microscope slide on a drop of bidistilled water. Slides were then left to dry for 4 hours on a heating platter (Medox, Germany) at 90 °C. The microscope slides with transverse cuts of nerve specimens were then stained with PPD stain.

As PPD stain primarily stains the myelin sheath of peripheral nerves, and myelinated axons are the functional axons of interest, this stain was deemed ideal for examining myelinated motor axons^26^. Microscope slides were left to dry, then covered with a cover slip using epoxy resin. The slides were then ready for light and digital microscopy.

### Image acquisition

Grayscale images were taken of each nerve Specimen transection at 200× (E-PL 10×/25 Br. foc. ocular and 20×/0.8; ∞/0.17 Zeiss EC Plan-Neofluar objective) magnification using a microscope (Zeiss Imager Z1) with a mounted camera (Zeiss Axio cam MR). Images were taken at lighting optimum of 3200 K using collaborating software (Axio Vision 4.8) on a computer system (Fujitsu-Siemens). The Images were then saved as tagged image files (.tif). Files could then be transferred to a personal computer.

### Image processing

Both manual counts and semi-automatic counts were carried out using Fiji software on an iMac (Late 2012) with macOS High Sierra software and a 2.7 GHz Intel Core i5 processor. The use of Fiji, a modified version of ImageJ, as public domain freeware^27^ was key to our method. This user friendly and research based freeware enables the research community to access a free image analysis tool with a multitude of plug-ins already included^27^.

Images were opened and processed as.tif files. Initial processing was common to both methods (manual and semi-automated) of nerve fibre analysis.

1. An image was opened with Fiji (File > Open > BiopsyXY.tif)
2. All nerve fascicles of the cross-section image were traced and cut (Tools > Polygon selections)
3. Then copied to a new clear image window (File > New > Image > file name = BiopsyXY_cut.tif, Typ = 8-Bit, Fill with = White).

**Figure 2:**
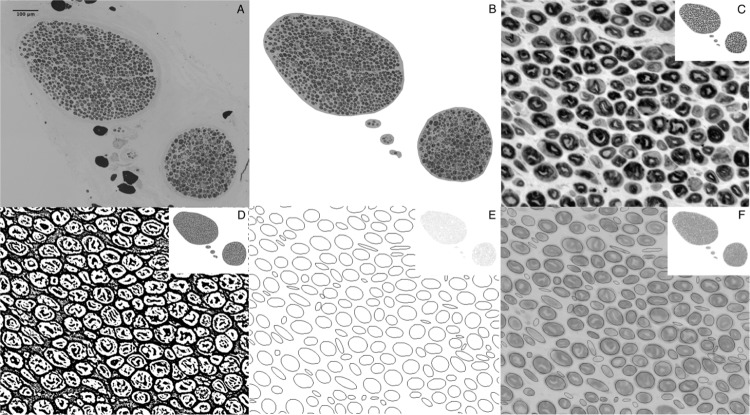
(**A**) Grey scale image, cranial zygomatic branch, 200x magnified, unprocessed. (**B**) Fascicles extracted (Background and artifact deletion) (**C**) Contrast enhancement with Fijis ‘CLAHE’ Local contrast enhancement function (**D**) ‘Auto Local Threshold’ – binary image (**E**)‚ Analyze Particles‘ (**F**) Overlay for demonstration purposes.

This first step eradicates the irregularities and artifacts of the background. The new image file contains only the corresponding nerve fascicles. (Fig. 2A,B)

### Manual counts

Manual counts were carried out by an independent blinded examiner involved in nerve sciences. The cut image was opened in Fiji (File > Open > BiopsyXY_cut.tif) and the cell counter tool selected (Plugins > Analyze > Cell Counter > Cell Counter). Next, ‘keep original’ checkbox was checked, ‘initialize’ was selected, a counter window opens, a counter type (Type 1) was selected, and counting commenced using a conventional computer mouse and a ‘count and click’ approach. When counting in larger images digital zoom was used to identify axons more accurately. The manual counting process was timed using a stop watch.

### Semi-automatic counts

Automatic particle detection was substantially modified by our group. The optimized calibration of settings and processing prior to counting included several steps.

1. The contrast of the grey scale image was set higher using Fijis ‘CLAHE’ function (Process > CLAHE (Enhance local Threshold) > Accurate).
2. Furthermore, the image was converted into a binary image using a uniform threshold adjustment (Image > Adjust > Auto Local Threshold > Method = Mean, Radius = 5, parameter_1 = 0, parameter_2 = 0).
  In the original image axons showed a darker grey shading than peri-, epi- and endoneurium. Using contrast enhancement and automatic thresholding with the ‘mean method’ allowed to produce a binary image, in which axons are enclosed white areas and background is black. (Fig. 2C,D)
3. The axons could then be automatically counted using specific settings with the analyze particle tool (Analyze > Analyze Particles…, size = 80–2500, circularity = 0.10–1.00, show = Ellipses) (Fig. 2D,E).

In order to further simplify this process, all settings and tools described in steps 1–3 in the ‘semi-automatic counts’ section were summarized in a macro (Plugins > Macros > Record… > Create > Save as AutoCount). Thus, all images could be processed automatically (Process > Multiple Image Processor) using macro ‘AutoCount’; a macro, which is freely available upon request. Axon counts were presented in an output table, which was copied and pasted to SPSS software for statistical analysis. The automated counting method macro was applied to all specimens using the Multiple Image Processor. The time taken for counting was recorded using a stop watch.

### Statistical methods

Statistical analyses were performed using SPSS (IBM Corp. Released 2016. IBM SPSS Statistics for Macintosh, Version 24.0. Armonk NY: IBM Corp.) and R (version 3.5.1; The R Foundation for Statistical Computing, Vienna, Austria). Bland-Altman plots were used to compare the automated counting method to the manual counting method (gold standard). Differences in axon counts were plotted against manual counts to show distributions and mean differences. 95% limits of agreement were calculated as mean difference ±1.96*standard deviation. The correlation between both methods was assessed by Spearman’s rho. Furthermore, the number of axons was dichotomized into <900 vs ≥900 axons for both methods as indicator for a good functional outcome. Here sensitivity defines the proportion of specimens correctly identified to have an axonal count ≥900 through semi-automated analysis, as previously determined by manual counting. Specificity measures the proportion of specimens found to have an axonal count <900 by semi-automated analysis, out of the total number of specimens that truly have less than 900 axons.

## Results

### Analysis time

Semi-automatic axon counting took 1 hour and 47 minutes for all 129 biopsies (average 50 seconds per biopsy, 0.04 seconds per axon). The counting process is automatic and must not be supervised. Manual counting took 21 hours and 6 minutes in total (average 9 minutes and 49 seconds per biopsy, 0.52 seconds per axon).

### Axon counts

**Figure 3:**
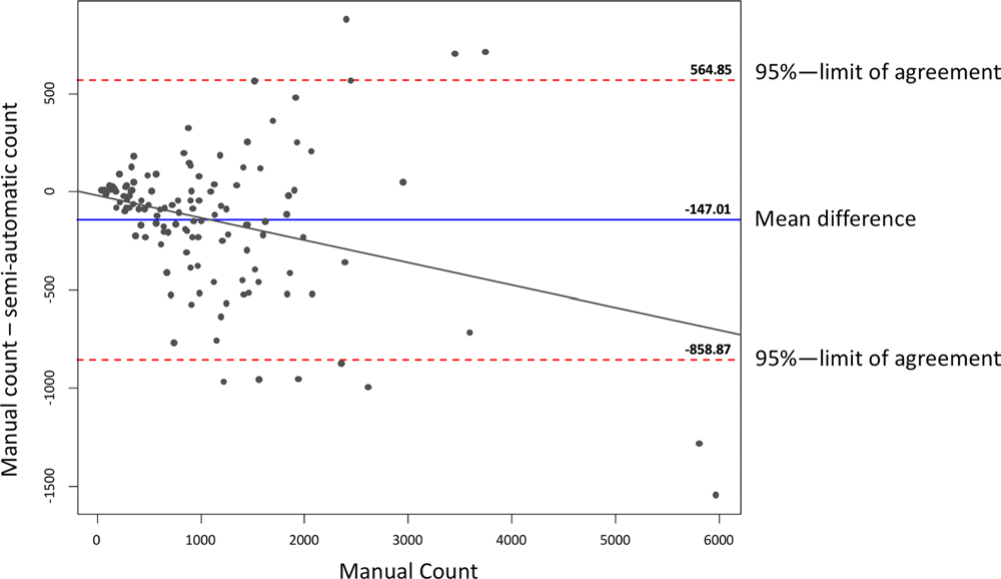
shows a Bland-Altman plot plotting the differences between the semi-automated and manual method on the y-axis against the manual counts as goldstandard values on the x-axis. In this depiction an outlier is removed for format purposes (−3832 axon difference, manual count 1894). The mean difference of automated and manual counts is −147 (blue line). Thus, the automatic method produces higher counts than manual. The range within the 95% limits of agreement is high (red dotted lines). The difference between counting methods increases with increasing absolute axon count (black regression line).

Figure 3 shows a Bland-Altman plot plotting the differences between the automatic and manual method, with manual counts being considered the goldstandard. A mean difference of −147 axons is shown. Thus, the automatic method produces higher counts on average than the manual method. The range within the 95%-confidence limit of agreement is high between −859 and 565. The difference between counting methods increases with increasing absolute axon count (see regression line).

**Figure 4:**
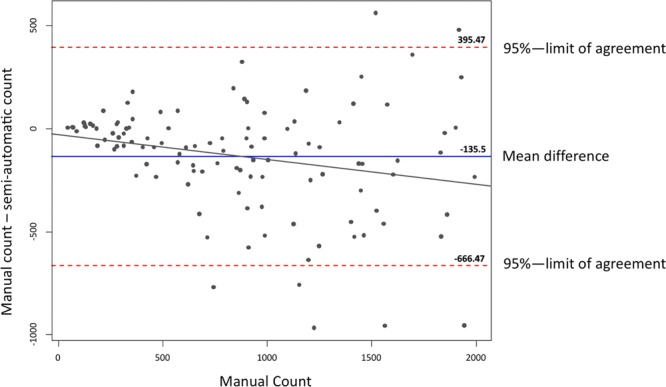
shows a Bland-Altman plot of axon counts up to 2000, representing the specific margin of interest. This figure shows clearly that in lower axon counts the methods match well, dispersing with higher counts. The mean difference between automated and manual counts is −136 (blue line). 95% limits of agreement are shown with red dotted lines, while the regression line is drawn as a black line.

Figure 4 shows a Bland-Altman plot of axon counts up to 2000, representing the specific margin of interest. This figure shows clearly that in lower axon counts the methods match well, dispersing with higher counts.

The novel semi-automated technique demonstrated a linear correlation to the manual method (Spearman rho = 0.944).

**Figure 5:**
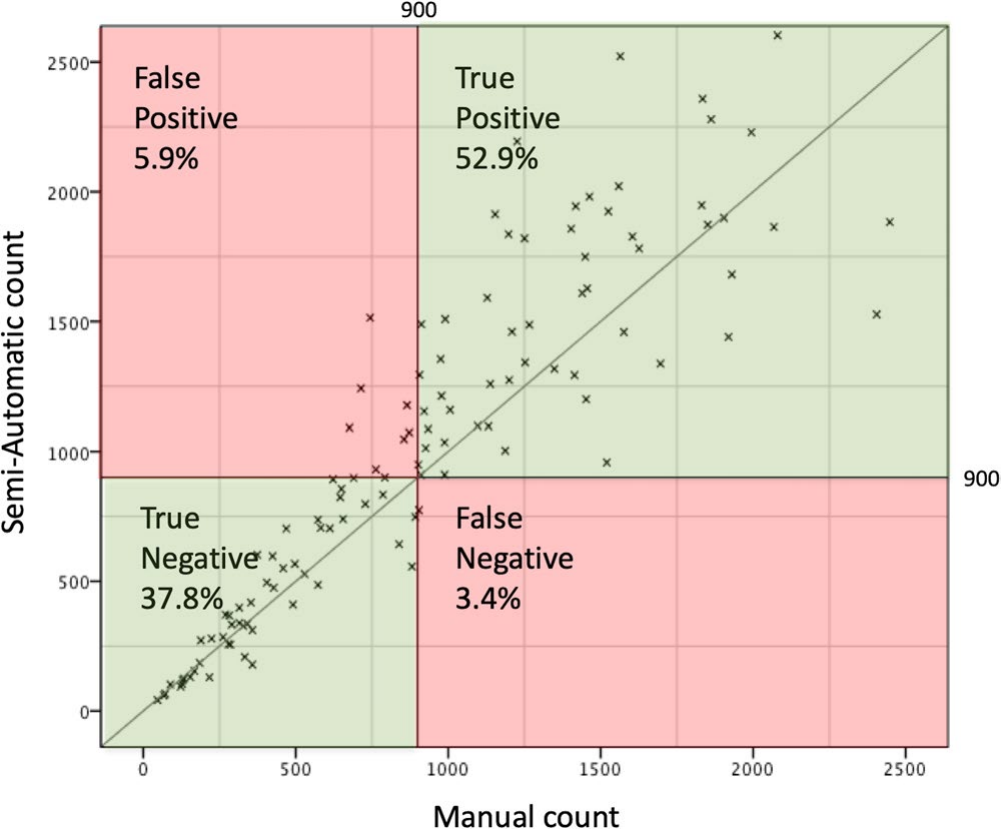
shows a scatter chart of all axon counts comparing manual and semi-automated methods. The cut off value of 900 axons is indicated as a vertical and horizontal line. Both green shaded areas show the counts of semi-automated and manual methods matching greater than 900 (top right), or lower than 900 (bottom left). Areas shaded red show specimens falsely identified as having more than 900 axons by the automated method (top left), or falsely identified as having less than 900 axons (bottom right).

For a correct identification of nerve biopsies with ≥900 axons the semi-automated method was found to be highly sensitive (94%) and specific (87%). The scatter chart in Fig. 5 shows that the semi-automated method is a reliable method in finding true positive (52.9%) (over 900 axons) and true negative (37.8%) (under 900 axons). Thus, the cut-off of 900 axons is determined correctly by the semi-automatic method in over 90%. High sensitivity and specificity are also guaranteed when determining other cut-off values, for example exceeding 1000 (sensitivity 98%, specificity 79%) or 1500 axons (sensitivity 87%, specificity 86%). Respective scatter charts can be found in the Supplemental Data Sheet.

## Discussion

A wide variety of histological axon quantification has been applied in the past. Existing methods include manual, semi-automatic and automatic approaches^7,28^.

Manual methods have proven to be tedious, time consuming and labor intensive. Furthermore manual methods may be associated with examiners fatigue and inconsistency^29^. However, manual methods allow for human correction and control whilst examining specimens of different quality. In certain cases observer interference is necessary^6,30^. This must be considered when opting for a fully automated method in nerve analysis.

Fully automated methods of nerve morphometry, more specifically in axon quantification are superior in that they are free of human input, subjective decision making and have faster processing speeds. Previous studies have shown average processing speeds of 1.5–10 s per analysed axon, when using fully automated methods^4,9^. Yet, accuracy of nerve analysis is adversely affected by higher processing speeds. The most common problem of fully automatic methods is their inability to discriminate between artifactual features or perineural tissue from actual axons. Blood vessels, blood cells and connective tissue may also be mistaken as nerve fibres in a cross-sectional cut^3,31^. Fully automatic methods range from highly specific and cost intensive approaches using immunostaining and fluorescent microscopy to simple and cost-efficient methods such as described by Tobin *et al.*^32,33^. Alternative semi-automatic methods exist in research groups with a high level of expertise and specialized apparatuses being able to produce a variation of data in histomorphometric analysis^8,34^.

Combining the best of both described approaches, a semi-automatic method allows for high processing speeds, good accuracy, high cost efficiency and manual alteration where necessary. The semi-automatic method presented in this study has a manual preparation phase in order to remove background and artifactual features and a fully-automated analysis phase. Axon quantification was significantly faster (p < 0.001) with 50 seconds per biopsy (time per axon 0.04 seconds) compared to 9 minutes and 49 seconds (time per axon 0.52 seconds) with the manual quantification method.

In our study PPD (paraphenylendiamine) stain was used for histologic preparation. PPD stain has the attribute of labeling the myelin sheath strongly^35^. Therefore we could ensure that primarily axons of interest were stained, allowing these to be counted by both the semi-automated and manual methods, while simultaneously ensuring a strong contrast between the myelin sheath and other structures prior to digital image preparation.

Whilst other studies used two different applications for image preparation and analysis, such as adobe photoshop and ImageJ, or Paint.net and ImageJ, this study utilized only one software for the entire process^3,32^. Urso-Baiarda and Grobbelaars approach showed many advantages opposed to traditional manual quantification methods^3^. However, imitating the multi-step method was found to be quite complex. The approach of alternating between different software may assure optimal features when adjusting and analyzing the images, however, our approach was to keep the method as simple as possible using only one freeware for adjustment and analysis of images. The advantage of the method described by Urso-Baiarda, is that the axons remain in their original size and stature during processing, making it possible to, not only determine axon count, but also axonal area, and perhaps with further analytics myelin area. Examining only axon counts, not needing to consider axonal area, myelin area or other parameters, allowed us to decisively simplify the method. The simplification of axon quantification in our study comes at a minimal cost of accuracy in absolute axon counts. The technique used by Urso-Baiarda *et al*. was very accurate with counts ranging from 98.7% to 106.1% of the reference count^3^. As shown in Fig. 2 our semi-automatic counts vary minimally from manual counts. The semi-automated method presented by Hunter *et al*. also shows high accuracy and precision when evaluating area and diameter of nerve features. However a specialized cost-intensive software (Leco morphometry Software) was used^11^. Freeware such as Fiji, ImageJ and Image editing software such as Paint.net and Adobe Photoshop is widely available and has shown to be accurate and sufficient in nerve analysis^3,32^. This can be confirmed by our study. As this method was developed as part of a facial and masseteric nerve study, examining peripheral branches, the axon count of examined specimens was mainly between 500 and 2000. Figure 4 shows a Bland-Altmann plot considering axon counts up to 2000. Within this margin the semi-automatic method counts axons with a mean difference of −135. Nevertheless, our method was also applied to cross sections of the facial nerve system that have higher axonal loads than 2000, such as the facial nerve main trunk and main arborization sites.

Our automated method counted an average axon capacity of 6684 ± 1884 (Range 2655–12457) in facial nerve main trunks (n = 87)^21^. This matches well with average axon quantities found in facial nerve main trunks in several other study groups (Hembd 5329 ± 1376, Captier 6490, Kondo 6245 ± 860)^36–38^.

In facial surgical cross-face-nerve graft procedures it is known that donor nerves with axon capacities exceeding 900 axons lead to good functional results^13,36^. Therefore key to our method was detecting the cut-off of 900 axons. The semi-automatic method was shown to be very precise in doing so (Fig. 5). Cut-off values of 1000 and 1500 axons where also investigated and found to be determined reliably. Our axon quantification comprised morphometry of the fifth cranial nerve at the level of the masseteric branch as well showing comparable counts (unpublished data). Axonal loads in brachial plexus surgery are similar to those seen in facial nerve surgery: nerves used for transfers showed 1318 (ulnar) and 1860 (median) axons respectively^20^. Donor nerves applicable for neurotization procedures in facial paralysis such as masseteric (1542)^39^ and spinal accessory (1054–1603)^40–42^ also demonstrated comparable axon count ranges. However, another significant nerve utilized in this field, the obturator nerve supplying gracilis muscle (free functional muscle transplants) was described with a significantly lower axonal load of 342^39^. As an important nerve for reconstruction the sural nerve was reported to comprise 1074 axons also showing a comparative number as an example for a relatively thick sensory nerve^14^. Thus, in summarization, our method should be applicable to determine the axonal load for all peripheral nerves of different fibre qualities with a high level of precision.

A simple and accessible method as used in this study may not be ideal to determine exact axon counts, but is reliable in determining quantity margins. The novel method shows larger inaccuracies with larger axon counts, however axon margins of interest are often within lower regions of axon capacities. In peripheral nerve surgery, especially facial nerve reconstruction, axon capacities of interest seldomly exceed 2000 axons^13,20,43^. In addition to this, manual counting methods may also regress in accuracy with larger axon quantities due to examiners fatigue.

A limitation to automatic and semi-automatic histological analysis is the quality of specimen cross section slides. Factors determining quality are extensive and include initial tissue quality during dissection, fixation, histologic processing, staining and image acquisition^4,7,28^. Certainly the aim is to keep these variables constant, however this is not always possible. Automatic and semi-automatic methods work with fixed algorithms, making it difficult to consider such variables. Nonetheless these variables also effect manual counts. The main limitation of our study seemed to be quality and freshness of the harvested biopsies, not the quantification method in itself. Large nerve specimens with large axonal loads often have fixation artefacts, hence absolute axonal loads may not be able to be determined as accurately.

Other recent studies on the microanatomy of the facial nerve with large research collectives have used semi-automated or automated methods for axon quantification^36,44^. Smaller research collectives in this field have been counted manually^14,16,38^. No uniform and gold standard method is used throughout different research projects. This makes it difficult to compare axon counts as well as to conduct reliable and reproduceable studies. Therefore, we propose a method, which is cost-efficient, user-friendly and reproduceable, ideal for axon quantification in peripheral nerves.

## Conclusion

Medical treatment of any kind performed on peripheral nerves requires a high degree of knowledge about their macro and microanatomy. Microanalysis of peripheral nerve cross-sections is used in basic research as well as clinical research for reasons named above. Axon quantification is an essential aspect in peripheral nerve surgery and is commonly achieved by either manual counts or highly specialized and expensive computer-based methods. Time expenses are often high when using fully automated axon analysis. Processing speeds of 1.5–10 s per analysed axon have been achieved in the past^4,9^. The semi-automated method presented here takes, on average, 0.04 s per axon, and is thus faster than fully automated or manual counting methods, including the control group in this study (0.52 s per axon). The method has been used to study the microanatomy of the facial nerve in 106 facial halves^21^.

The newly introduced method simplifies precise, rapid axon quantification of peripheral nerves with a freely available ImageJ software - Fiji. Reliable analysis of micrographs is tremendously accelerated by computer-based batch processing. Convenient computer analysis is possible on any conventional laptop. Axon analysis itself is thus not bound to a laboratory setting. Processing speed may be improved even further using high-end computers with high-speed processors. The proposed freely available semi-automated method greatly reduces time expenses made on accurate and reproducible micrograph analysis.

The necessary screening for nerves with more than 900 axons can reliably be carried out with this method, whilst still maintain high levels of specificity and sensitivity. In regard to the cut-off value of 900 axons it is very specific and sensitive.

## Supplementary information

Supplementary Information - Method Protocol.

## Data Availability

The datasets generated during and analysed during the current study are available from the corresponding author on reasonable request.
