# Author Correction: Rapid and Precise Semi-Automatic Axon Quantification in Human Peripheral Nerves

**DOI:** 10.1038/s41598-020-63860-5

**Published:** 2020-04-17

**Authors:** S. Engelmann, M. Ruewe, S. Geis, C. D. Taeger, M. Kehrer, E. R. Tamm, R. L. A. W. Bleys, F. Zeman, L. Prantl, A. Kehrer

**Affiliations:** 1 Department of Plastic, Hand and Reconstructive Surgery, University Hospital Regensburg, Regensburg, Germany; 2 Department of Trauma Surgery, University Hospital Bonn, Bonn, Germany; 3 Institute of Human Anatomy, University of Regensburg, Regensburg, Germany; 4 Department of Anatomy, University Medical Center Utrecht, Utrecht, The Netherlands; 5 Center for Clinical Studies, University Hospital Regensburg, Regensburg, Germany

Correction to: *Scientific Reports* 10.1038/s41598-020-58917-4, published online 06 February 2020

The Article does not include a reference to a recent relevant paper, which is now cited as Reference 1 below, and should be included in the text as follows.

In the Discussion,

“Large nerve specimens with large axonal loads often have fixation artefacts, hence absolute axonal loads may not be able to be determined as accurately.

Other recent studies on the microanatomy of the facial nerve with large research collectives have used semi-automated or automated methods for axon quantification.”

should read:

“Large nerve specimens with large axonal loads often have fixation artefacts, hence absolute axonal loads may not be able to be determined as accurately.

The ultimate goal of microanatomic nerve research is optimizing intraoperative decision making. Other studies have attempted intraoperative axon quantification successfully in animal nerve cross sections using a rapid automated quantification method, however being dependent on commercial software [1].

Other recent studies on the microanatomy of the facial nerve with large research collectives have used semi-automated or automated methods for axon quantification.”
