# Supplementary material for: Rapid and Precise Semi-Automatic Axon Quantification in Human Peripheral Nerves
Source: Sci Rep. 2020 Feb 6;10:1935. doi: 10.1038/s41598-020-58917-4 (PMC7005293; doi:10.1038/s41598-020-58917-4)

Rapid and Precise Semi-Automatic Axon Quantification in Human Peripheral Nerves.

Supplementary Information.

Engelmann S[1], Ruewe M [1], Geis S [1], Taeger C[1], Kehrer M[2], Tamm ER[3], Bleys R.L.A.W[4], Zeman F[5], Prantl L[1], Kehrer A[1]

[1] Department of Plastic, Hand and Reconstructive Surgery, University Hospital Regensburg/Germany
[2] Department of Trauma Surgery, University Hospital Bonn/Germany
[3] Institute of Human Anatomy, University of Regensburg/Germany
[4] Department of Anatomy, University Medical Center Utrecht/The Netherlands
[5] Center for Clinical Studies, University Hospital Regensburg, Germany

Protocol

Semi-automated Axon Quantification

Applications of the method

Our protocol for axon quantification in peripheral nerves can be used on any nerve biopsy cross sections examining axons with myelin sheath. The myelin sheath is essential in the staining with PPD and automated analysis process. Good use of the analysis method is expected with specimens counting up to 2000 axons. In larger nerve biopsies inaccuracies may occur due to a higher chance of artefacts and lower quality of histologic processing in large biopsies. Batch processing during axon analysis enables a large cohort of specimens to be analyzed.

Materials

Reagents

The reagents listed below are those utilized in our laboratory and can be substituted by similar commercially available reagents from other suppliers.

**Reagent**	**Supplier**
Acetone	Merck, Darmstadt, Germany
Azure II	Merck, Darmstadt, Germany
Cacodylic acid Sodium-salt $3H_2O$	Serva Electrophoresis GmbH, Heidelberg, Germany
DDSA (Dodecenyl succinic anhydride)	Serva Electrophoresis GmbH, Heidelberg, Germany
DMP-30 (tri-dimethylaminomethyl phenol)	Serva Electrophoresis GmbH, Heidelberg, Germany
Ethanol	Roth, Karlsruhe, Germany
Glutaraldehyde (Caution! Toxic, wear appropriate chemical protective gloves.)	Serva Electrophoresis GmbH, Heidelberg, Germany
Glycidyl ether	Serva Electrophoresis GmbH, Heidelberg, Germany
Methylene blue	Merck, Darmstadt, Germany
MNA (Methyl nadic Anhydride)	Serva Electrophoresis GmbH, Heidelberg, Germany
Sodium tetraborate	Merck, Darmstadt, Germany
OsO_4 (Osmium tetroxide) (Caution! Toxic, wear appropriate chemical protective gloves.)	Science Services, München, Germany

Paraformaldehyde (Caution! Toxic, wear appropriate chemical protective gloves.)	Merck, Darmstadt, Germany
para-phenylendiamine	Roth, Karlsruhe, Germany

Reagent Setup

**Reagent**	**Setup**
0.2 M Sodium cacodylate buffer	0,2 M Sodium cacodylate solution (Serva, Heidelberg) (42,8g/1000 ml)<br><br>with 0,2 M HCl to be titrated to ph 7,4
Glutaraldehyde 25%	Solution in water
Paraformaldehyde (PFA) 25% (Caution! PFA is highly toxic and large amounts of vapors are released during preparation. Always use in a fume hood.)	25g Paraformaldehyde in 100 ml bidistilled water
Osmium ferrocyanide	Solution A: 2% watered OsO_4 (Science Service, München)<br><br>Solution B: 1,6% watered Potassiumhexacyanoferrat-II (Merck, Darmstadt) (96 mg/6ml),<br><br>in 0,2 M Sodium cacodylate buffer<br><br>Solutions A and B are to be mixed 1:1
EM-Fixation stock solution	10 ml Paraformaldehyde 25%, 50 ml 0.2 M Sodium cacodylate buffer, 30ml bidistilled water, (pH 7,4)
EM-Fixation	9 ml EM-Fixation stock solution + 1 ml glutaraldehyde 25%
Richardson stain	

Equipment

**Equipment**	**Supplier**
Embedder	EM TP Leica, Wetzlar, Germany
Inlab pH electrode	Mettler Toledo, Giessen, Germany

Leica EM Trim2	Leica, Wetzlar, Germany
Diamond milling head	E. Friedl & Co. Industriediamanten, Meß- und Regeltechnik GmbH, Wien, Austria
Specimen holder	Leica, Wetzlar, Germany
Universal lab oven	Memmert, Schwabach, Germany
Knifemaker 7800	LKB, Bromma, Sweden
Glass 8mm	Science Services, München, Germany
KL 1500 LCD (Stereo microscope)	Zeiss, Oberkochen, Germany
Mettler AE 163 micro balance	Mettler Toledo, Giessen, Germany
Microscope Axio Imager.Z1	Zeiss, Oberkochen, Germany
Zeiss Axio Cam MR	Zeiss, Oberkochen, Germany
Fujitsu-Siemens PC	Fujitsu Siemens Computers GmbH, München, Germany
iMac (Late 2012) with macOS High Sierra software	Apple Inc., Cupertino, California, USA
pH-meter	Knick, Berlin, Germany
Microtome - 2218 Historange	LKB, Bromma, Sweden
Vacuum Pump	BOC Edwards, Crawley, West Sussex, Great Britain
Histo diamond knife	Diatome, Switzerland
Heating plate	Medax Nagel GmbH, Kiel, Germany
Slide cover glass 24x60mm	Menzel-Gläser, Braunschweig, Germany
Pasteurplast pipettes	VWR, Darmstadt, Germany
Powderfree Sempercare laboratory gloves	Sempermed, Wien, Austria
Super Frost® Slides	Menzel-Gläser, Braunschweig, Germany
Razor Blade Personna	American Safety Razor Company, Verona, USA
Glass products	Schott AG, Mainz/ Carl Roth GmbH & Co. KG, Karlsruhe/ VWR Darmstadt, Germany

Software

**Software**	**Supplier**
Axio Vision 4.8	Zeiss, Oberkochen, Germany
Fiji	Freeware

Procedure

Nerve fibre analysis in our study is structured into 3 main steps: Histological preparation, Image acquisition and lastly image analysis using our refined method for semi-automated axon quantification. A total of 1238 nerve biopsies were analyzed using this Protocol.

Histological processing

Fixation and Embedding (Timing 2 d)

1) Take fresh nerve biopsies and place in labelled Eppendorf tubes with EM-Fixative solution (2.5% formaldehyde, 2.5% glutaraldehyde) for 12 hours to rest.
2) Wash biopsies with 0.1 M Sodium cacodylate buffer at pH 7,4. Let biopsies sit in buffer for 30 minutes.
3) Repeat step 2 four times.

The Biopsies are then osmicated in an automated embedder. Steps 4-10 are carried out in the EM TP Leica Embedder.

4) For post fixation processing set the biopsies into osmium ferrocyanide solution for 2 hours and 30 minutes at +4°C.
5) Wash biopsies, resting them in bidistilled water for 30 minutes.
6) Repeat step 5 four times.
7) Set biopsies into bidistilled water over night.
8) The samples are then serially dehydrated in alcohol and impregnated with epoxy resin using the embedder and following scheme:

50%	EtOH	2x15min	4°C
70%	EtOH	2x15min	4°C
80%	EtOH	2x15min	4°C
90%	EtOH	2x15min	20°C
Abs.	EtOH	2x15min	20°C
EtOH/ Aceton 1:1		15min	20°C
Aceton		2x15min	20°C

9) The Biopsies can now be embedded using following scheme:

Mixture Aceton: EPON

• 3:1	1h	20°C
• 2:1	1h	20°C
• 1:3	1h	20°C

10) The embedded biopsies are now polymerized in the laboratory oven for two days at 60°C. These can then be removed from the rubber moulds and are ready for histologic sectioning.

Processing semi-thin microscope slides (Timing 1 d)

11) Take an embedded biopsy in its Epon block and clamp it into a specimen holder. Now mill down excess Epon using Leica EM Trim2 with the diamond milling head, until a transverse section of the biopsy is at the surface of the block. (*Troubleshooting)
12) Clamp the specimen holder including the specimen into a stereo microscope. Under vision prepare the block into a pyramidal shape using the razor blade.
13) The Specimen holder is now to be clamped in the microtome for initial cutting. Initial cutting is to be carried out using a glass knife. The microtome is set at 2μm.
14) Place initial 2μm cuts onto a glass slide using a fine forceps and stain with Richardson stain for fast evaluation of the cut. This slide is for quality control and evaluation of positioning of the embedded biopsy. Later it will be discarded.
15) View the Richardson stained cut under the stereo microscope to ensure correct layer and positioning of cut. (*Troubleshooting)
16) Replace the glass knife with a diamond microtome knife. These come with a trough, which is to be filled with bidistilled water. Set microtome to 1μm for final semi-thin cutting.
17) Cut 16 cuts for each biopsy, using a fine brush to lift each semi-thin section floating on the water trough and place onto a glass slide (8 each). This is most easily done by placing a drop of bidistilled water onto the glass slide initially. Produce 16 cuts per Biopsy, distributed on 2 microscope slides. The semi-thin cuts can be placed onto the drop of water. This step is critical in obtaining optimal cross-sections. Sections must be cut by a trained employee and are to be handled with care.
18) Place the microscope slides with the semi-thin cross sections onto a heating platter for at least 2 hours at 90°C. This ensures flattening and adhesion to the glass slides.
19) Now place slides into PPD- Stain for 30 minutes.
20) Rinse slides by immersing into ethanol for a few seconds.
21) Cover slides with a cover glass and Epon to make slides durable and long-lasting.
22) Dry microscope slides over night in laboratory oven at 60°C.

Image Acquisition (Timing 5 hours)

23) Use the digital microscope Axio Imager Z1 combined with the Zeiss greyscale Axio cam MR and a Zeiss EC Plan-Neofluar 5x/ 0,15; ∞/ 0,17' Objective to inspect a microscope slide of the chosen biopsy. Find an appropriate cut of the specimen.
24) Now switch to the Zeiss Plan-APOCHROM AT 20x/ 0,8; ∞/ 0,17' Objective for detailed inspection.
25) Lighting optimum is set to 3200K. For most cuts the area is too large to be digitalized in one shot. Thus the 'mosaic' function of the Axio Vision 4.8. software is used to record the specimen as a whole.
26) Mosaic recording must be adapted with following settings:
    a. „Stitching":
        - Search depth: „15"
        - Minimal overlap: „3"
        - Sobel operator: „low"
        - Stitching-Mode: „Stitching-position"
        - Reference channel: „1"
        - Reference Z-position: „1"
        - Reference time: „1"
        - Reference time utilization: „yes"
    b. „Convert tiles":
        - Zoom: „1"
        - Brightness compensation: „yes"
        - Copy tile information: „No"
        - EnableInputImageROI: „No"
27) The images are saved as 'Carl Zeiss Vision Image' (.zvi) automatically. Select file and convert to 'Tagged Image Files' (.tif). This step can be done after digitalization process of all specimens as stack conversion.

Semi-automated axon quantification

Manual adjustment (Timing 1 hour)

28) Transfer Image files to any appropriate Personal Computer. In this Study an iMac (Late 2012) with macOS High Sierra 10.13 software was used.
29) Open 'Fiji' Software
30) Open the original grey scale image of the desired nerve biopsy transection. (File > Open > ImageXY.tif)

31) Open a new image file and set window size appropriate to the original image. In this new file the adjusted image will be placed in the next steps. Name the file as desired, in this case 'ImageXY_cut.tif' (File > New > Image > Image=ImageXY_cut.tif, Typ=8-Bit, Fill with=White, Width= Set according to ImageXY.tif, height= Set according to ImageXY.tif)
32) Use the selection tool to cut the nerve fascicles in the original image and paste them into the new image file. This way all background artefacts are eliminated. (Tools > Polygon selections > Cut nerve fascicles in ImageXY.tif and paste into ImageXY_cut.tif). Images must be cleared of background in order for the automated analysis to work correctly.
33) Save ImageXY_cut.tif

Automated Processing (Timing 10 mins)

The following steps are comprised into a macro function and must not be carried out singularly. Batch processing is possible for axon analysis. Nonetheless the single steps are listed for clarity.

34) Open the desired adjusted image (ImageXY_cut.tif)
35) Use following Fiji functions to adjust and modify the image qualities. Process > CLAHE (Enhance Local Contrast) > Accurate. This function is an automatic contrast enhancement.
36) In the original image axons show a darker grey shading than peri-, epi- and endoneurium. Using contrast enhancement and automatic thresholding with the mean method allows to produce a binary image, in which axons are enclosed white areas and background is black. (Figure 1 C and D) Image > Adjust > Auto Local Threshold > Method=Mean, Radius=5, parameter_1=0, parameter_2=0.
37) Finally the image can be analyzed, counting axons automatically. Analyze > Analyze Particles. Adjust the following settings: size=80-2500, circularity=0.10-1.00, show=Ellipses. Axons are analyzed and counted. Results are shown in the output data table. (*Troubleshooting)

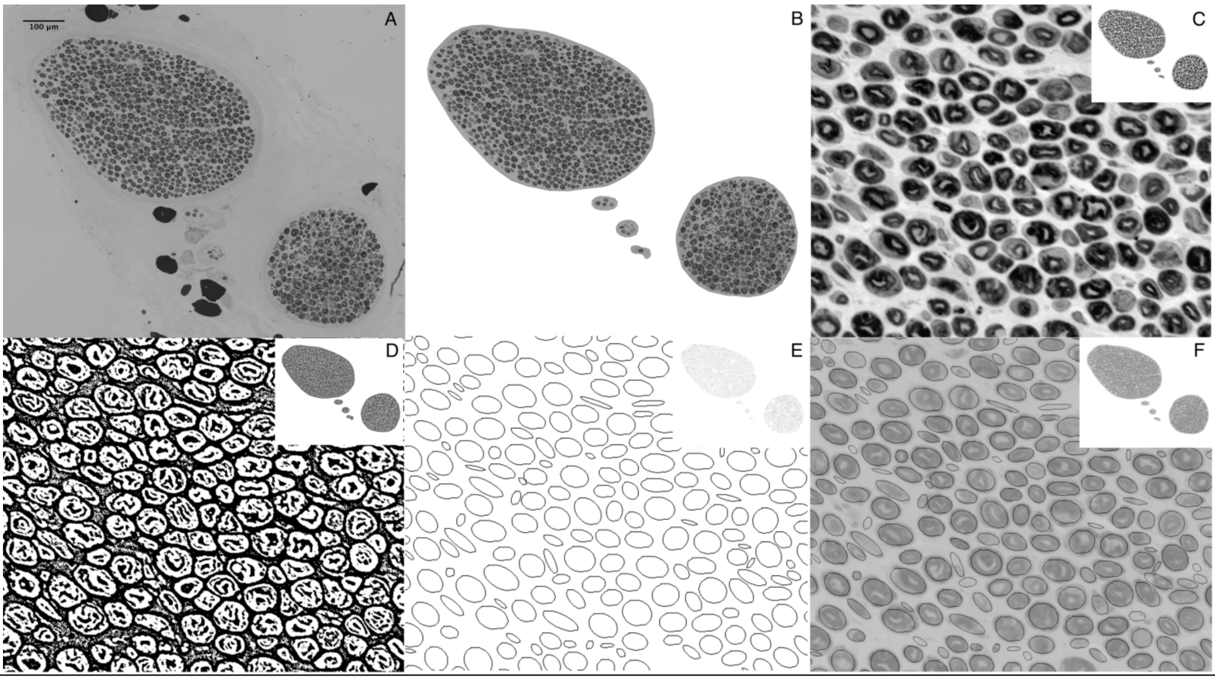

Figure 1: A) Grey scale image, cranial zygomatic branch, 200x magnified, unprocessed. B) Fascicles extracted (Background and artifact deletion) C) Contrast enhancement with Fijis 'CLAHE' Local contrast enhancement function D) 'Auto Local Threshold' – binary image E) ‚Analyze Particles' F) Overlay for demonstration purposes.

Timing

The total time needed and time needed under active supervision vary greatly, due to several long resting times and automated processes. Also, times vary according to amount of specimens examined, however it is possible to process large batches of specimens depending on the step. For instance a stack of over 1000 images can be processed in one batch automatically in steps 34 – 37. The Timing listed below is an example based on approximately 30 biopsies.

Step	Description	Timing	Explanation
1-10	Fixation and embedding	2 days	These steps include resting times and processing I the embedder. Work time carried out by laboratory staff is ~2 hours
11-22	Processing semi-thin slides	1 day	These steps are labor intensive and require trained laboratory staff for histologic slide preparation.
23-27	Image acquisition	5 hours	Timing for image acquisition is highly dependent on practice of the staff member. With good routine a single specimen can be digitalized in up to 5 minutes instead of 10 minutes as stated here.
28-33	Manual Image adjustment	1 hour	1 Specimen needs ~2 mins for manual adjustment.
34-37	Automated image analysis	10 mins	The personal computer needs a certain amount of time to process the images. This

			however can run in the background and does not require supervision. Therefore time required for laboratory staff is merely a few seconds to initiate stack processing

* Troubleshooting

Step	Problem	Possible reason	Solution
11	When milling the epon block, specimens may suffer cracks	Milling down epon too fast and too large steps	Handle milling machine with care and progress into epon block with smaller steps
15	Specimen is not transversely cut	Specimen may have curled up during osmication and embedding or was simply set into block diagonally	Inspect block under microscope and adjust positioning in block holder. Repeat milling ad cutting process
37	Axon counts are not congruent with control counts ie. Manual counts.	Several reasons may lead to this problem. Good quality of cross sections is needed. All axons must be recognizable and identifiable. Quality of cross sections may be altered during fixation, histologic processing, less probably during image acquisition.	Exact axon counts may vary to control counts. A margin of error must be accounted for. When encountering larger deviations, histologic processing should be repeated under strict quality control.

Scatter Charts

Scatter Chart 1 Showing Cut-off axonal load of 900 determined by Semi-automated Method
Sensitivity: 94%
Specificity: 87%

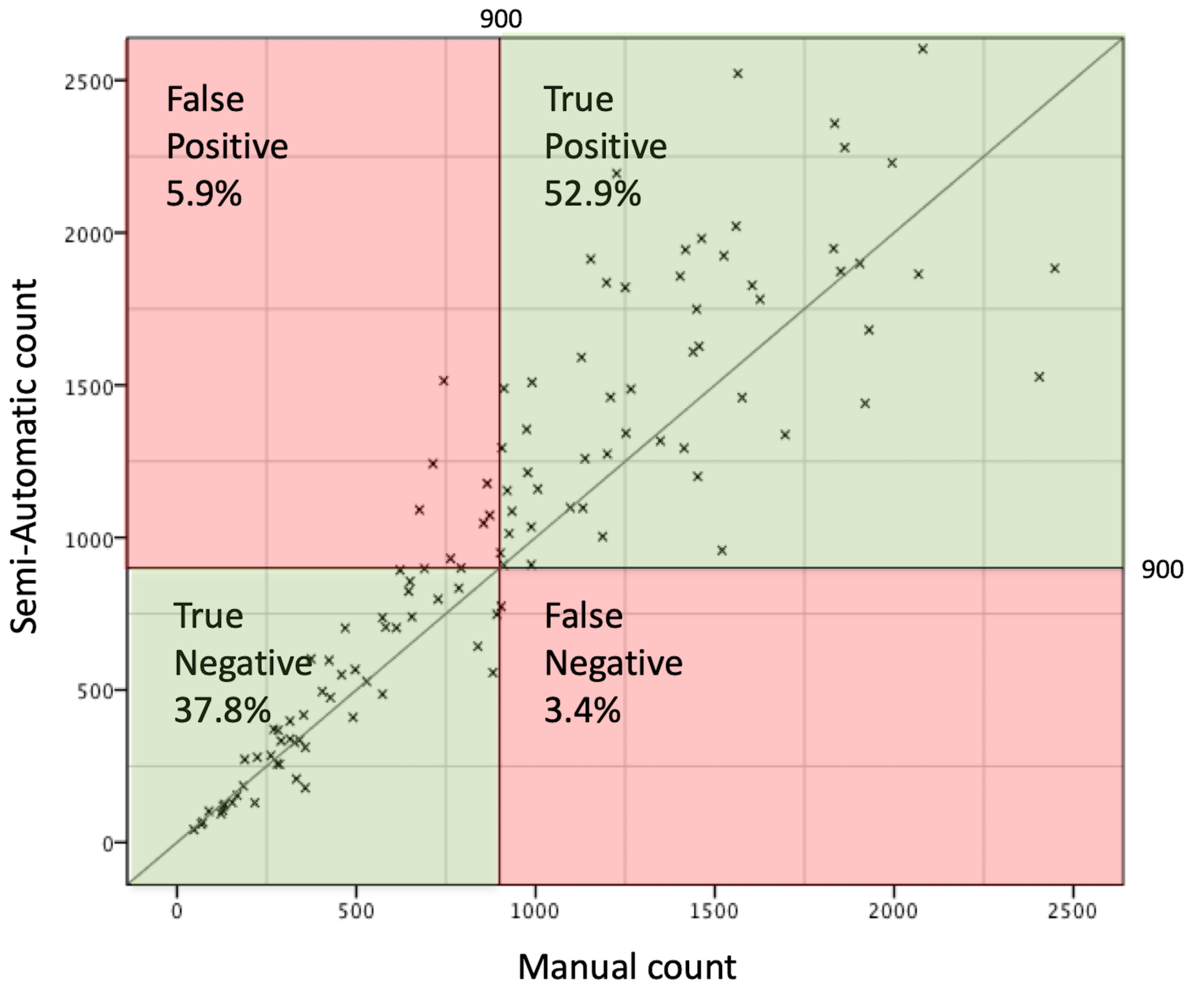

Scatter Chart 2 Showing Cut-off axonal load of 1000 determined by Semi-automated Method
Sensitivity: 98%
Specificity: 79%

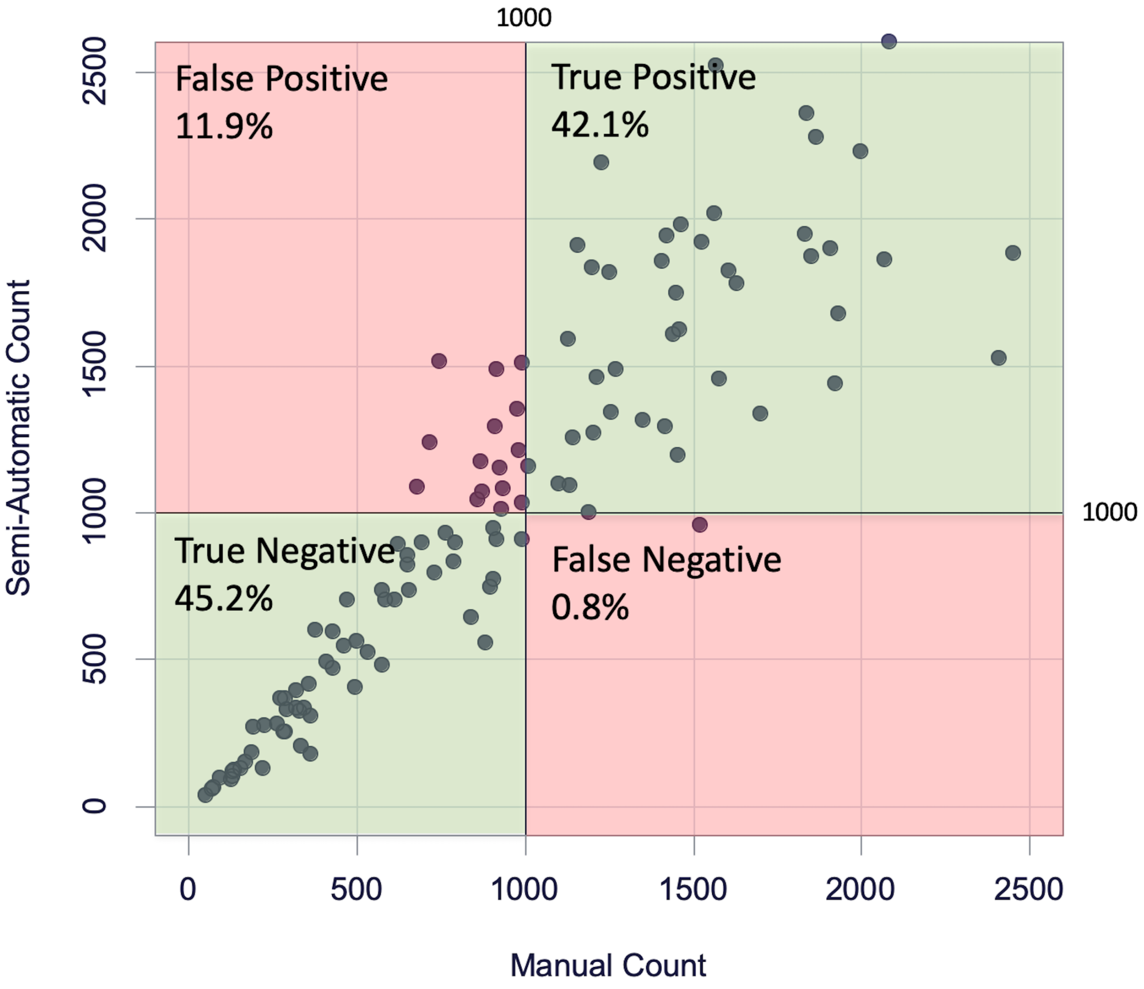

Scatter Chart 3 Showing Cut-off axonal load of 1500 determined by Semi-automated Method
Sensitivity: 87%
Specificity: 86%

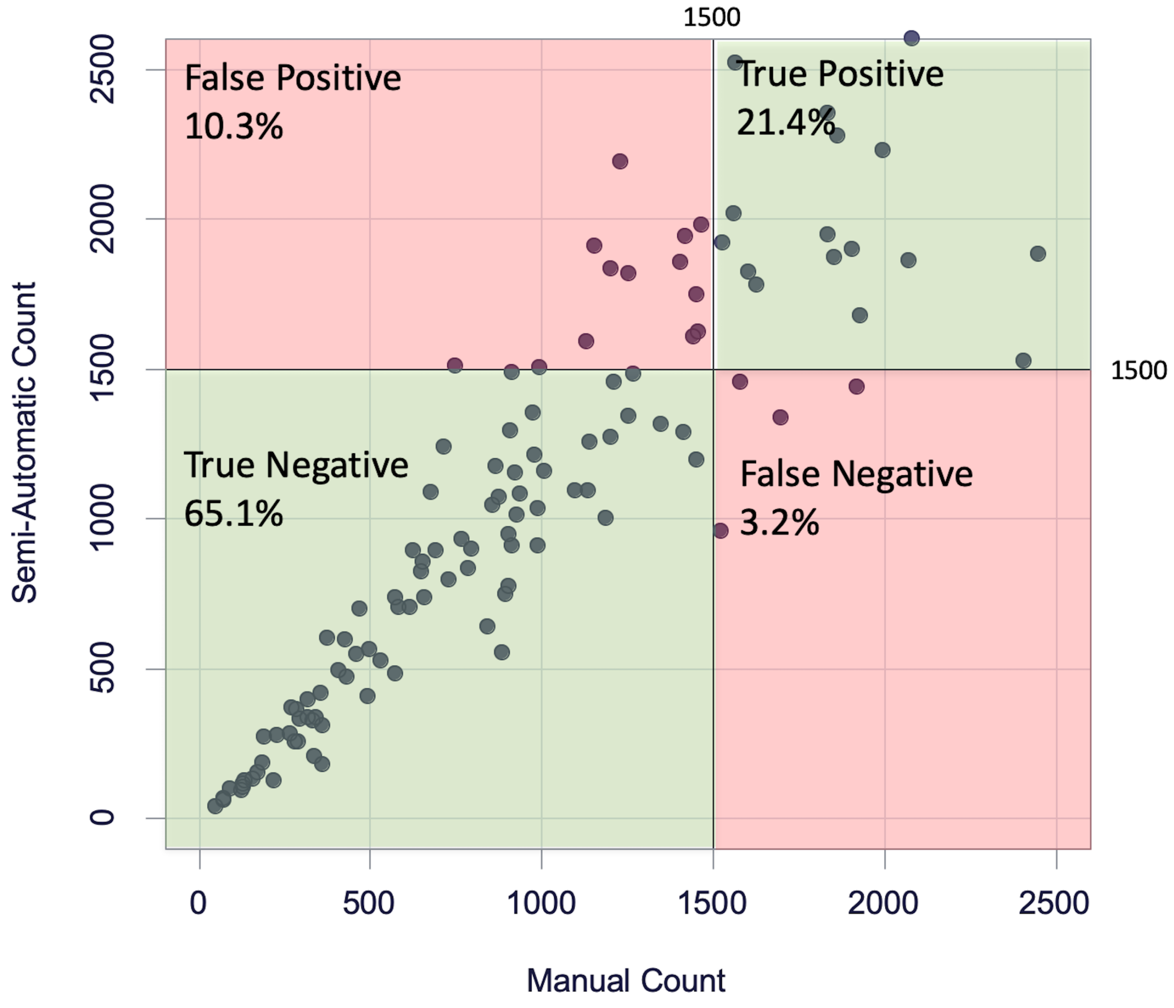

Supplement: Supplementary file 1 — Supplementary Information - Method Protocol. [file 41598_2020_58917_MOESM1_ESM.pdf]
